# Healthcare Hackathons Provide Educational and Innovation Opportunities: A Case Study and Best Practice Recommendations

**DOI:** 10.1007/s10916-016-0532-3

**Published:** 2016-06-08

**Authors:** Julie K. Silver, David S. Binder, Nevena Zubcevik, Ross D. Zafonte

**Affiliations:** Department of Physical Medicine and Rehabilitation, Harvard Medical School, Spaulding Rehabilitation Hospital, 300 1st Avenue, Charlestown, MA 02129 USA; Physical Medicine and Rehabilitation, Massachusetts General Hospital, Boston, MA 02114 USA; Physical Medicine and Rehabilitation, Brigham and Women’s Hospital, Boston, MA 02115 USA

**Keywords:** Hackathon, Interdisciplinary, Healthcare training, Education, Rehabilitation, Innovation

## Abstract

Physicians and other healthcare professionals are often the end users of medical innovation; however, they are rarely involved in the beginning design stages. This often results in ineffective healthcare solutions with poor adoption rates. At the early design stage, innovation would benefit from input from healthcare professionals. This report describes the first-ever rehabilitation hackathon—an interdisciplinary and competitive team event aimed at accelerating and improving healthcare solutions and providing an educational experience for participants. Hackathons are gaining traction as a way to accelerate innovation by bringing together a diverse group of interdisciplinary professionals from different industries who work collaboratively in teams and learn from each other, focus on a specific problem (“pain point”), develop a solution using design thinking techniques, pitch the solution to participants, gather fast feedback and quickly alter the prototype design (“pivoting”). 102 hackers including 19 (18.6 %) physicians and other professionals participated, and over the course of 2 days worked in teams, pitched ideas and developed design prototypes. Three awards were given for prototypes that may improve function in persons with disabilities. 43 hackers were women (42.2 %) and 59 men (57.8 %); they ranged in age from 16 to 79 years old; and, of the 75 hackers who reported their age, 63 (84 %) were less than 40 years old and 12 (16 %) were 40 years or older. This report contributes to the emerging literature on healthcare hackathons as a means of providing interdisciplinary education and training and supporting innovation.

## Introduction

Physicians and other healthcare professionals are often the end users of medical innovation; however, they are rarely involved in the beginning design stages. Too often this results in ineffective and inefficient solutions with poor adoption rates. Innovation efforts would likely benefit at the design stage from clinical experience and key insights from frontline healthcare professionals, particularly those at academic medical centers.

Hackathons, which bring together stakeholders in the early design phase, are becoming an increasingly popular way to identify the most urgent or important clinical needs and create new products, systems, services, datasets and tools that will improve healthcare delivery. Hacakthons and their cousins, datathons [1], have been recommended as open-source or “crowd source” models to support cross-disciplinary collaboration [2] in an effort to foster innovation in medicine [3]. Boudreau and Kakhani described four models of crowd-sourcing innovation in an article for the *Harvard Business Review* [4]. These models included: 1. a crowd contest (e.g., hackathon); 2. a crowd collaborative community (businesses teaming up with online communities that contain customers as well as software engineers and others); 3. a crowd complementor (e.g., developers that create complementary products for the iPhone); and, 4. a crowd labor market (third party intermediaries that match buyers and sellers). The first model, a crowd contest such as a hackathon, was deemed the most straightforward way to engage a crowd. Crowd contests work by identifying a specific problem, offering a prize and inviting people to submit solutions. These types of contests have been going on for centuries and have resulted in many scientific breatkthroughs.

Recently, Youm and Wiechman described the Med AppJam as a model for educating students and engaging them in technology healthcare solutions [5, 6]. Zaaijer and Erlich described using “applied hackathon sessions” in an academic classroom setting for undergraduate and graduate students to help educate them about genomics [7]. Students in architecture, design, enginnering, communication and anthropology participated in a hackathon in an effort to designed “youth friendly” hospital rooms. [8] Craddock et al. described a “brainhack” in which they used components from hackathons, unconferences and parallel educational sessions [9]. We propose that hackathons themselves, regardless of whether there are additional educational sessions offered, provide an opportunity to not only educate students but all participants, irrespective of age or area of expertise, via a unique process that supports interdisciplinary collaboration through open innovation. Despite the interest and growth of healthcare hackathons, currently there are very few published reports describing them in the medical literature.

The word hackathon is derived from *hack* and *marathon*. As the name suggests, the event involves an intense and discrete period of collaboration. Hackathons are not intended to be a standalone innovation event or process but rather are one part of an innovation continuum that can help accelerate and improve future solutions.

One of the challenging parts of starting a hackathon is trying to explain what this is to healthcare professionals who are unfamiliar with the term. The literature offers various descriptions but no clear definition [10]. Because of the confusion we encountered when trying to describe a healthcare hackathon to various internal and external individuals who we needed support from (e.g., hospital administrators) or wanted to encourage to participate (e.g., physicians), we quickly recognized the need to more clearly articulate what it is. Therefore, we have developed a new definition as follows:

*A****healthcare hackathon****is a competitive event (live or virtual) that has three specific goals--accelerating the innovation of medical solutions, improving the design in the beginning stages, and supporting educational training for all participants--and aims to accomplish them by focusing on a specific problem (pain point), bringing together in an open innovation format (internal and external resources) an interdisciplinary group of individuals (hackers) that include, but are not limited to, physicians and other healthcare professionals, data scientists, engineers, user interface designers, business professionals, students and other stakeholders who work in teams and follow a process to develop initial prototypes, pitch them to a panel of judges experienced in innovation and quickly alter them according to the feedback (pivoting).*

We have also summarized some of the commonly used terms that participants in hackathons should be familiar with (Table 1).

**Table 1:** Commonly Used Terms in Hackathons

***Accelerator:*** A process whereby specific support (e.g., office space, internet access, legal advice, etc.) is provided to a start-up company in an effort to accelerate growth and product/solution development; sometimes the term accelerator is used to refer to a person who might provide mentorship and/or other support (e.g. financial) ***Design techniques:*** Best practices design theory and practice which supports the development of innovative products and solutions that end-users can afford and will use ***End-user:*** Healthcare professionals or patients that use the product or solution ***Hacker:*** A participant in the hackathon ***Hacking:*** The process of developing potential solutions at a hackathon ***Incubator:*** Often used synonymously with accelerator, although incubators often provide support for longer periods of time; incubators provide startups with shared or co-working space and sometimes exposure to other startup companies (e.g. exposure to the startup ecosystem) ***Medtech:*** Technology solutions that are specific to the healthcare space ***Mentor:*** Advisors from various sectors who provide guidance during the hackathon ***Open innovation:*** A process whereby both internal and external resources are used ***Pain Points:*** Problems healthcare professionals or patients encounter ***Pipeline:*** A healthcare business’s or organization’s list of products or solutions that are available or being developed ***Pitch:*** A hacker’s or hacker team’s brief presentation of a problem to hackathon participants or judges that typically is from 1 to 3 min and may include specific information such as competitive products or solutions, key differentiators in what is proposed and a business plan ***Pivoting:*** The process of quickly altering a prototype, during the hackathon itself, based on feedback	

## Approach

### Hackathon strategy

Our hackathon was a competitive interdisciplinary team event that aimed to accelerate and improve healthcare solutions for the rehabilitation patient population. Our goals included conducting the first-ever rehabilitation hackathon, sharing information with our colleagues about hackathons and how they might accelerate and/or improve innovation in rehabilitation medicine, developing prototypes for new products or services that support our patient population, engaging our own faculty and hospital staff in educational training around healthcare innovation and developing best practices for future healthcare hackathons. Determining the hackathon strategy is a critical part of planning the actual event. We began our strategy with the basic theme of hosting a hackathon that was focused specifically on rehabilitation medicine and the problem or “pain point” of needing to improve the health and/or ability to function of persons with disabilities. To our knowledge, this is the first hackathon dedicated to rehabilitation medicine innovation and the post-acute care population. Next we worked directly with the Massachusetts Institute of Technology (MIT) Hacking Medicine team. To gain firsthand experience we participated in the MIT Grand Hack which is one of the largest health hackathons in the world. In addition to the hackathon experience, we referenced resources such as the online “Healthcare Hackathon Handbook” published by MIT Hacking Medicine [11].

### Pre-hackathon planning

With a hackathon, planning may result in not only a better experience for the participants but also enhance the development of technology prototypes.

Our planning began with choosing a theme that would support the acceleration of technology driven solutions in rehabilitation medicine for individuals with disabilities. Some healthcare hackathons have very narrow themes. For example one business school hosted a hackathon that was diagnosis specific for stroke and used the “time is brain” concept with an aim to innovate solutions to avoid delays in stroke diagnosis and/or treatment and prevent subsequent brain injury (of note is that this event was not focused on rehabilitation or the post-acute care population) [12]. Because to our knowledge this was the first hackathon focused on rehabilitation medicine, we opted to keep our theme broad and simply used “rehabilitation medicine” as the theme.

The timing and venue were also carefully selected to coincide with the 2015 Annual Assembly for the American Academy of Physical Medicine and Rehabilitation (AAPM&R) – this was a separate event that we leveraged to engage out of town colleagues, including physicians specializing in PM&R (physiatrists). This was also an event where there would be numerous announcements to physiatrists about the Annual Assembly that could incorporate information about the hackathon. So, the conference provided an opportunity to widely announce the hackathon to potential participants prior to the event and then also brought a large pool of potential participants to town. As we were recruiting many engineers, designers, and computer scientists from surrounding universities, it was also important to take into account undergraduate and graduate exam schedules.

Other planning was similar to what is required for events in general and included registration, parking, food, audio visual equipment and so on.

### The hackathon event

Day 1 involved setting up and registering the participants (hackers), judges, mentors and other volunteers. There were more than 150 people who attended with 102 registered hackers. The event began with a welcome and keynote speech followed by a “Hack 101” lecture that was designed to quickly educate attendees who were novice hackers. Next came the first pitching session by the hackers followed by mingling and team formation. Teams formed organically based on mutual interests and complementary skill sets. Once teams had formed, the remainder of Day 1 was spent discussing potential solutions and strategies (i.e. hacking). To help advance solutions and to offer guidance, more experienced hackathon mentors were available to answer questions or to offer advice. Typically, mentors are accomplished or experienced physicians, entrepreneurs or engineers.

By Day 2, the teams were formed, and hacking continued from the previous night. The morning was spent hacking (the teams working together, getting input from mentors, pivoting and refining their upcoming pitches). The final pitches were mid-afternoon, with a period for the judges to deliberate and then the awards ceremony and concluding remarks.

## Outcomes

**Fig. 1:**
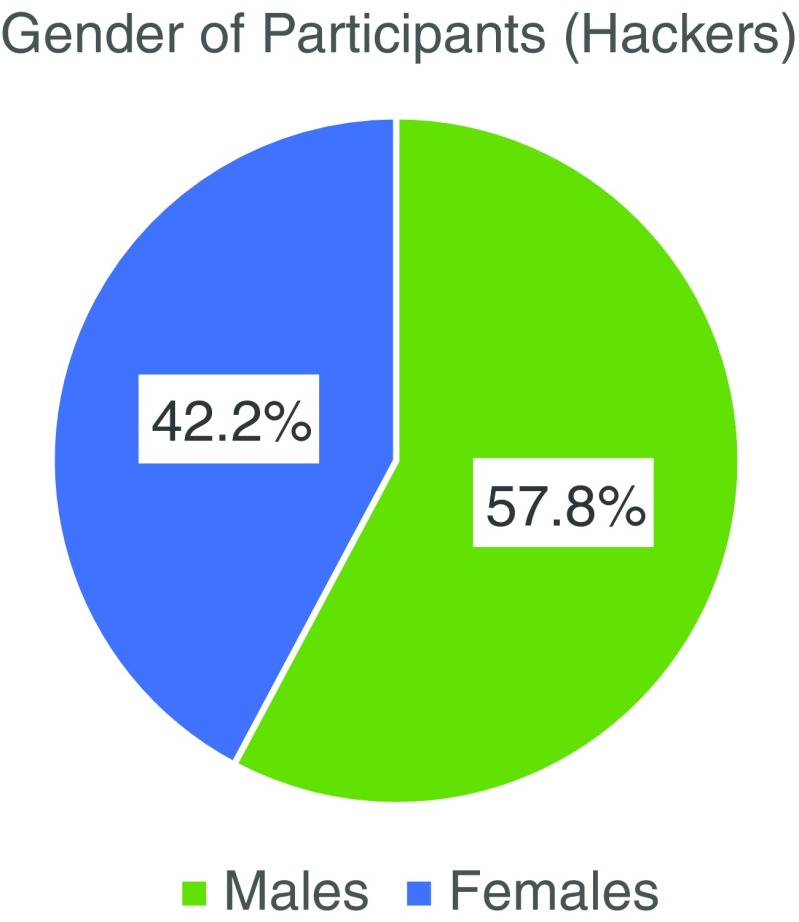
Legend: There were more men than women who participated (*n* = 102 total participants; 59 men and 43 women)

**Fig. 2:**
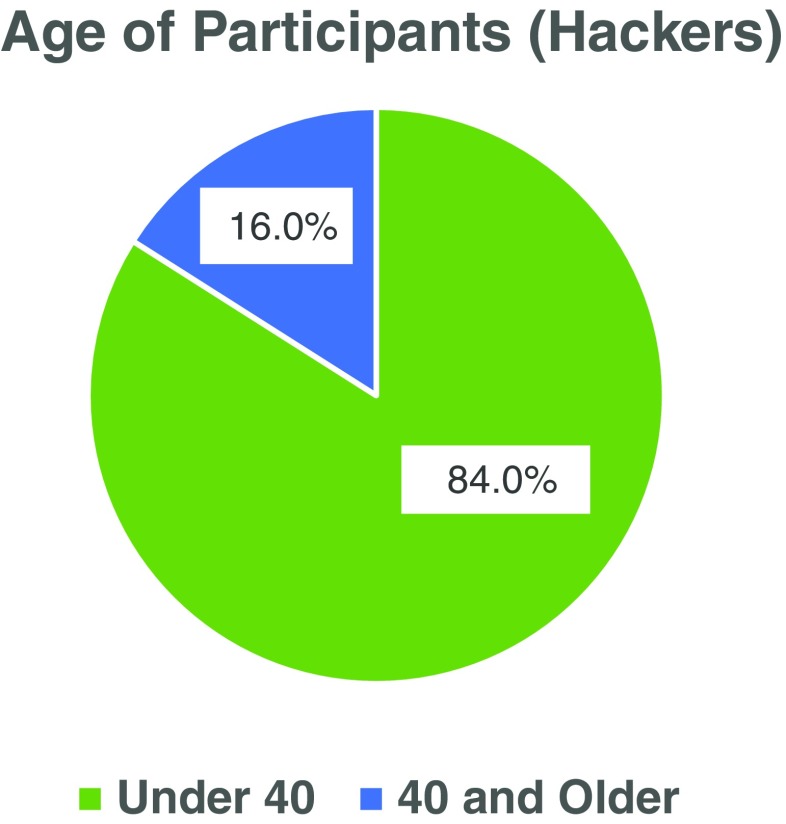
Legend: The majority of participants who reported their age (*n* = 75) were less than 40 years of age

**Fig. 3:**
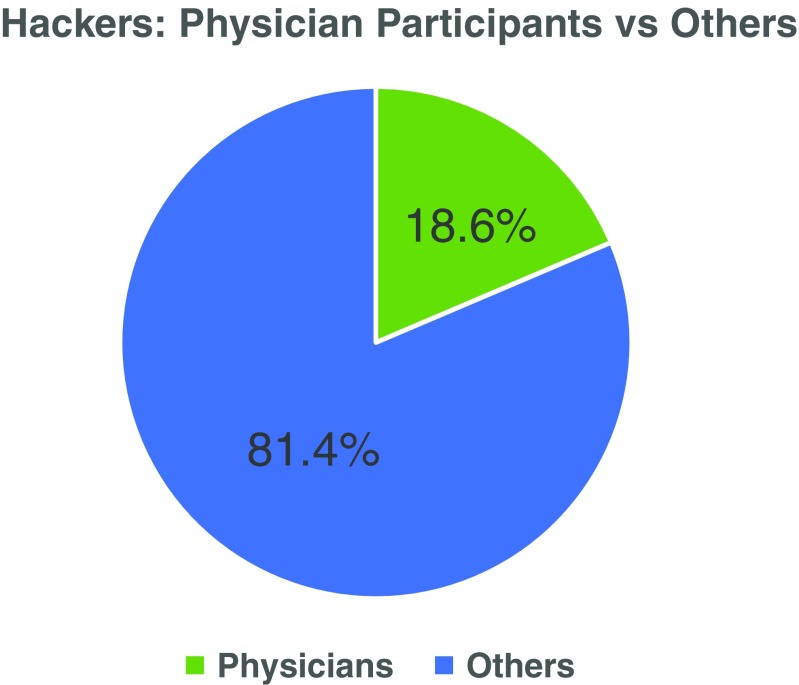
Legend: Nearly one out of five participants was a physician (*n* = 102 total participants)

Of the 102 hackers, there were 43 women (42.2 %) and 59 men (57.8 %) (Fig. 1). The hackers ranged in age from 16 to 79 years old. Of the 75 hackers who reported their age, 63 (84.0 %) were less than 40 years old and 12 (16.0 %) were 40 years or older (Fig. 2). Physicians made up 18.6 % (*n* = 19) of the total hackers and ranged in age from 24 to 64 years old with only two physicians reporting their age as 40 years or more (one was 40 years old and the other 64 years old) (Fig. 3). The other 81.4 % of the hackers consisted of a mix of engineers, designers, coders, scientists, non-physician clinicians, entrepreneurs, students and others. All of the participants were from the United States with the exception of two physicians who came from France and Qatar.

The 102 participants (hackers) formed 10 teams, and three awards were given. Judges decided on a 1st and 2nd place along with a “best design” award. The prizes were intended to support and promote further development of solutions.

## Implications

The Spaulding Rehabilitation Hackathon accomplished our initial goals and also led to interesting new developments. Our goals included conducting the first-ever rehabilitation hackathon, educating colleagues about hackathons and how they might accelerate and/or improve innovation in rehabilitation medicine, developing prototypes for new products or services that support our patient population, engaging our faculty and hospital staff in technology and innovation training and developing best practices for future healthcare hackathons (Table 2). Going forward, this will be an annual event and planning for the next one is already underway. Future hackathons may involve a series of focused conclaves to deal with domain specific disability issues.

**Table 2:**
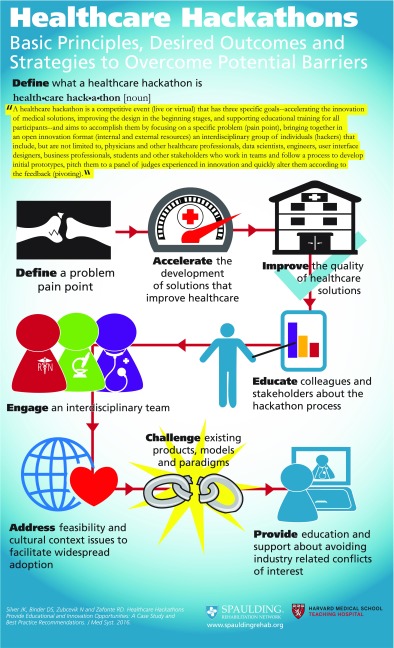
Infographic: Healthcare Hackathons: Basic Principles, Desired Outcomes and Strategies to Overcome Potential Barriers

Analysis of our hackathon revealed interesting demographic data. We also recognized an opportunity to collect additional data at future hackathons. Perhaps one of the best metrics for success is to measure how many teams continue to work on their solution after the event. For example, determining the number of original teams that are still working together at 3 months or 6 months post-hackathon. Another measure is financial support that may include post-event grants or other funding. Table 3 lists some proposed metrics for determining the success of a healthcare hackathon.

**Table 3:**
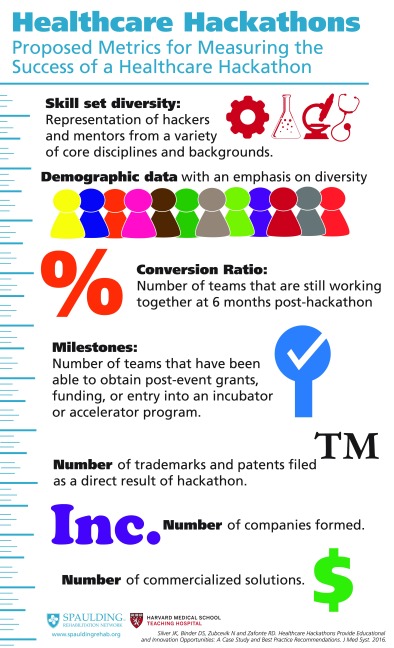
Infographic: Proposed Metrics for Measuring the Success of a Healthcare Hackathon

However, the data we collected for this hackathon demonstrated a need to encourage physicians and other healthcare experts over the age of 40 years to participate. Healthcare hackathons would benefit from the wisdom and experience that these individuals would bring and simultaneously support them in learning more about the development of technology solutions. Although typically in medicine it is the older generations mentoring the younger ones, hackathons (and perhaps some other methods of innovation, too) would likely benefit from incorporating strategies that include the mentoring of experienced clinicians by those they usually teach and mentor. We plan to test some strategies that we hope will encourage experienced physicians and other healthcare professionals to participate in greater numbers. Additionally, although the gender discrepancy was not as striking as the age discrepancy, going forward, we will also focus on encouraging more female physicians and other professionals to participate.

We also recognized an opportunity to share more information with physicians and other providers in our academic department and hospital regarding how best to navigate any potential or future conflicts of interest. By their very nature, hackathons involve industry, and this is a topic that requires careful navigation, particularly by those in academia. One of the greatest benefits of a hackathon is to bring together interdisciplinary teams to work on innovative solutions. Indeed, Walker and Ko stated that the “ongoing participation by health care providers is essential for the careful development, implementation and evaluation of any technological invention.” [13] However, this also may pose the greatest risk to healthcare professionals, particularly academic physicians, if these relationships continue beyond the event and involve any type of compensation. Although physician participation in hackathons is encouraged, and may be essential for producing the optimal solutions, it is important to help support them in navigating the challenges associated with industry. It is worthwhile reviewing departmental and institutional disclosure and conflict of interest guidelines and protocols and working directly with the experts on this at your institution. Refer to Table 4 for a list of best practices.

**Table 4:** Best Practices for Healthcare Hackathons

1. Define what a healthcare hackathon is to internal and external stakeholders so that they understand this and are able to provide the needed support. 2. Educate internal and external stakeholders about the hackathon process using a common language (taxonomy). 3. Identify a theme and/or define the problem (pain point) that needs to be solved. 4. Choose a time and a venue that will best support the event. 5. Invite hackers, sponsors, collaborators, etc. with a genuine interest in the hackathon’s goals. 6. Prepare written agreements that describe the expectations and deliverables for all participating organizations. 7. Select metrics, collect data and analyze the results of the hackathon. 8. Anticipate concerns, particularly from academic physicians and scientists, about the potential for conflicts of interest and assist them in avoiding these. Identify relevant policies on intellectual property and distribute to all participants.	

One unanticipated and notable opportunity that came about shortly after our first hackathon was that Spaulding’s Dean Center for Tick Borne Illness was invited to join forces with the White House Office of Science and Technology Policy in designing the first Lyme Disease Hackathon. This hackathon is aligned with the White House Precision Medicine Initiative and Invisible Illnesses efforts.

Healthcare hackathons are quickly evolving and provide unique training opportunities for all participants. This report describes the first-ever rehabilitation hackathon, offers a new definition for the term *healthcare hackathon*, proposes metrics for measuring the success and benefits of hackathons, and suggests a list of best practices for physicians and other medical professionals to consider if they decide to conduct or participate in one. Hackathons have the potential to bring about positive change through “disruptive innovation”. They bring together people from diverse backgrounds for the common purpose of creating solutions to healthcare related pain points. In doing so, they also provide a platform for education, collaboration, integration, and the acceleration and/or enhancement of innovative solutions.
